# Evaluation of Maternal Immunization Rates in Primary Care: A Retrospective Study From Selected Family Health Centers in İstanbul, Türkiye

**DOI:** 10.3389/ijph.2026.1609233

**Published:** 2026-03-17

**Authors:** Saliha Büşra Aksu, Seda Özmen Sever, Yağmur Gökseven Arda, Güzin Zeren Öztürk

**Affiliations:** 1 Şişli Hamidiye Etfal Education and Research Hospital, Istanbul, Türkiye; 2 Şişli Hamidiye Etfal Education and Research Hospital, TC Saglik Bakanligi Basaksehir Cam ve Sakura Sehir Hastanesi, Başakşehir, Türkiye

**Keywords:** antenatal care, immunization programs, maternal immunization, pregnant women, primary health care

## Abstract

**Objectives:**

To assess maternal vaccination coverage in primary care and to examine its association with completion of antenatal care visits and maternal characteristics in Türkiye.

**Methods:**

A retrospective record-based observational study including 996 pregnancies registered at Family Health Centers in Istanbul (2020–2022) was analyzed. Vaccination records for tetanus–diphtheria (Td), tetanus–diphtheria–acellular pertussis (Tdap), hepatitis B, influenza, and coronavirus disease 2019 (COVID-19) were retrieved from electronic health records and the National Vaccination Tracking System. Women attending all four scheduled antenatal care visits were classified as having complete antenatal care. Descriptive analyses, chi-square tests, and binary logistic regression analysis were performed.

**Results:**

Overall, 89.7% of pregnant women received at least one vaccine. Complete vaccination rates were 79.7% for Td/Tdap, while complete hepatitis B immunization status was observed in 9.8%, and complete COVID-19 vaccination in 10.7%. Two-thirds (66.4%) completed antenatal care visits. Complete antenatal care was independently associated with higher odds of vaccination during pregnancy (aOR = 2.91, 95% CI: 1.91–4.44), having a lifetime immunization record (aOR = 3.12, 95% CI: 1.74–5.61) and complete Td/Tdap vaccination (aOR = 2.16, 95% CI: 1.57–2.98).

**Conclusion:**

Maternal vaccination coverage in Türkiye remains below international targets except for Td/Tdap. Greater continuity of structured antenatal care and continuity of care visits may be associated with higher maternal vaccination uptake. In addition integrating all recommended maternal vaccines into national protocols may support improvements in immunization coverage.

## Introduction

Pregnancy is a period of increased vulnerability to infectious diseases due to physiological and immunological adaptations that ensure fetal tolerance while maintaining maternal defense [1–4]. Consequently, vaccine-preventable infections such as influenza, pertussis, hepatitis B, and COVID-19 can lead to serious maternal and neonatal complications, including hospitalization, preterm birth, and neonatal mortality [5–7]. Maternal immunization-defined as the administration of selected vaccines during pregnancy to protect both the mother and the newborn-has been demonstrated to be a safe and cost-effective public health strategy and is endorsed by major international authorities, including the World Health Organization (WHO), the Centers for Disease Control and Prevention (CDC), and the American College of Obstetricians and Gynecologists (ACOG) [8–10].

According to these international guidelines Tdap, influenza, and COVID-19 vaccines are recommended during every pregnancy, while other vaccines such as hepatitis A, hepatitis B, meningococcal, pneumococcal, and respiratory syncytial virus (RSV) vaccines are considered in the presence of specific risk factors, based on clinical evaluation by the physician [8–10]. Similarly, in Türkiye, national recommendations largely align with global guidelines. During the COVID-19 pandemic, the Ministry of Health recommended vaccination against COVID-19 during pregnancy, and the vaccines were made available within the national immunization program; however, as of 2022, COVID-19 was no longer included in routine recommendations. Currently, tetanus toxoid–containing (Td/Tdap) vaccines and the influenza vaccine are recommended during every pregnancy. Hepatitis B vaccination is not part of the routine schedule during pregnancy but is recommended for women of reproductive age and for those identified as being at risk based on clinical assessment [11, 12].

In Türkiye, maternal immunization is integrated into national antenatal care protocols regulated by the Ministry of Health. The Antenatal Care Management Guide and the Family Medicine Implementation Regulation stipulate that pregnant women should attend at least four antenatal care visits in FHCs [13, 14]. All recommended maternal vaccines are provided free of charge at Family Health Centers (FHCs), where family physicians and nurses routinely evaluate immunization status during these antenatal care visits.

Despite these structured programs and free vaccine access, vaccination uptake during pregnancy remains suboptimal in Türkiye. Previous studies in Türkiye have mainly focused on general vaccine hesitancy or postpartum vaccination behaviors [15, 16], with limited data evaluating vaccination coverage during pregnancy within the context of primary care. Moreover, few studies have investigated how the completeness of antenatal care—a key component of family medicine practice—is associated with vaccine uptake in real-world settings, despite international evidence indicating that structured antenatal care visits are associated with higher maternal vaccine acceptance [17–19].

Therefore, this study aimed to evaluate maternal vaccination coverage among pregnant women attending FHCs in Istanbul between 2020 and 2022, and to examine its association with completion of recommended antenatal care visits, focusing on Td/Tdap, hepatitis B, influenza and COVID-19 vaccines.

## Methods

### Study Design and Setting

This retrospective record-based observational study was conducted between January 1, 2020, and December 31, 2022, at all eight family medicine units operating within two Family Health Centers affiliated with the Şişli Hamidiye Etfal Family Medicine Clinic, located in the Sultangazi district of Istanbul, Türkiye. These centers were included using a facility-based convenience sampling approach. Together, the participating Family Health Centers provide primary care services to approximately 26,100 registered individuals, including about 7,425 women of reproductive age.

The sampling frame consisted of all pregnancies registered and followed within the participating FHCs during the study period and completed their pregnancies by December 31, 2022. Consecutive inclusion was applied, and no random sampling or additional selection procedures were used.

Pregnancies ending in stillbirth or abortion, as well as cases with missing or incomplete data, were excluded. During the study period, a total of 1,174 pregnancies were recorded across FHCs. After excluding 58 pregnancies that did not result in live birth and 120 pregnancies for which antenatal care visit information could not be consistently retrieved due to changes in family health center during pregnancy, a total of 996 pregnancies were included in the final analysis. Although vaccination data for these excluded pregnancies were available through the National Vaccination Tracking System, incomplete documentation of antenatal care visits and selected sociodemographic or obstetric variables—depending on the timing of transfer during pregnancy—precluded reliable classification for analysis. The pattern of missing data was therefore predominantly related to continuity of care factors rather than random data loss.

Women with more than one pregnancy during the study period were included with each pregnancy analyzed separately. Each pregnancy was analyzed as an independent observation because vaccination and antenatal care visit records were specific to each pregnancy. Only 48 women (4.8%) had more than one pregnancy during the three-year period, suggesting a low likelihood of meaningful within-subject correlation.

**FIGURE 1:**
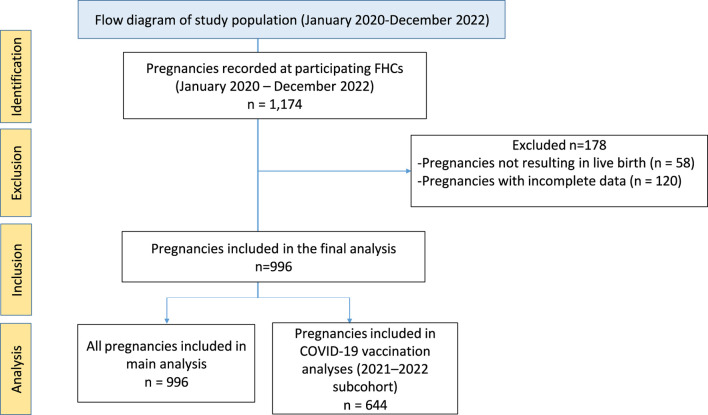
Flow diagram of pregnancy selection for the study conducted at Family Health Centers (FHCs) in Istanbul, Türkiye The diagram illustrates the identification, exclusion and inclusion of pregnancies followed at participating Family Health Centers between January 2020 and December 2022, as well as the analytic cohorts used for the main analyses and the COVID-19 vaccination subcohort. Evaluation of maternal immunization rates in primary care: a retrospective study from family health centers in Istanbul, Türkiye, 2020–2022.

All statistical analyses were performed on these 996 pregnancies with complete vaccination data. However, because COVID-19 vaccines became available in Türkiye only in 2021, analyses related to COVID-19 vaccination were restricted to pregnancies completed in 2021 and 2022. (Figure 1).

### Data Collection and Variable Definition

Following ethics committee approval, data extraction was conducted by researchers between May and June 2024 using the electronic health record systems of the participating Family Health Centers and the National Vaccination Tracking System.

All Family Health Centers are formally registered within the national health information infrastructure and have authorized access to national systems for individuals registered to their units. The electronic systems used in Family Health Centers are fully integrated with the National Vaccination Tracking System; therefore, vaccinations administered at Family Health Centers, as well as those provided by other healthcare institutions across Türkiye, are centrally recorded and accessible through the national system.

Data linkage was performed at the individual level using the unique national identification number assigned to each patient. Vaccination records were retrieved from both the Family Health Center electronic records and the National Vaccination Tracking System and were cross-validated. In cases of discrepancies, the National Vaccination Tracking System was considered the reference source. Although the national system provides near-complete coverage, vaccinations documented only on personal vaccination cards and not entered into electronic systems may not have been captured.

Data extraction and analysis were performed by researchers with authorized access to the Family Health Center electronic systems. Prior to analysis, basic data quality checks were applied, including assessment of duplicate records, internal consistency of vaccination dates, and compatibility of vaccination timing with pregnancy periods.

Demographic and clinical characteristics-including maternal age, age at first pregnancy, gravidity, parity, number of abortions, number of living children, pregnancy-related complications, and the presence of chronic diseases, were recorded.

According to the Antenatal Care Management Guide in Türkiye [13], four routine antenatal care visits are scheduled during pregnancy at FHCs. In this study, completion of antenatal care visits was determined based on documentation of all four scheduled visits recorded in the structured antenatal module of the Family Health Center electronic health record system. Women who attended all four visits were classified as having completed the recommended antenatal care visits. This variable was used as an indicator of continuity of antenatal care within primary care settings. Hospital-based antenatal care visits were not systematically captured in this study, as the primary objective was to evaluate continuity of antenatal care and vaccination practices within the primary care setting.

Data were collected on tetanus–diphtheria–acellular pertussis (Tdap), tetanus–diphtheria (Td), hepatitis B (HepB), influenza, and COVID-19 vaccines. In Türkiye, Tdap was recommended for pregnant women but was officially included in the National Immunization Program after 2025 [12]. Therefore, although Tdap data were recorded, the numbers were insufficent for meaningful statistical analysis. For this reason, Tdap was not analyzed separately and was evaluated together with Td vaccines. Other vaccines such as hepatitis A, meningococcal, pneumococcal, and RSV were not included in the analysis, as they are recommended only for selected risk groups and infrequently recorded in the study population.

Vaccine-specific definitions and completeness criteria were based on national maternal and adult immunization guidelines issued by the Turkish Ministry of Health and aligned with international recommendations from the World Health Organization and the Centers for Disease Control and Prevention [8, 9, 11, 12]. Complete vaccination was defined as the administration of all doses recommended for each vaccine in the national immunization schedule. Participants who had received fewer than the required doses were classified as incompletely vaccinated.

Vaccination status was determined based on the following criteria [8, 9, 11, 12]:

#### Tetanus Toxoid-Containing Vaccines

For consistency, tetanus vaccination refers to any tetanus toxoid–containing vaccine (Td or Tdap) throughout the manuscript. Complete Td/Tdap vaccination was defined as receipt of at least two doses of a tetanus toxoid–containing vaccine during the current pregnancy, or documentation of at least two doses in a previous pregnancy plus a booster dose during the index pregnancy. During the study period, the vast majority of tetanus toxoid–containing vaccines administered during pregnancy consisted of Td, as Tdap was not routinely available within the national immunization program.

#### Hepatitis B

Complete hepatitis B immunization status was defined as documentation of a full three-dose hepatitis B vaccine series and/or anti-HBs seropositivity recorded in the national vaccination registry. This definition reflects immune status rather than confirmed vaccination history alone.

#### Influenza

Complete influenza vaccination was defined as receipt of the influenza vaccine during the evaluated pregnancy.

#### COVID-19

Complete COVID-19 vaccination was defined as receipt of at least two doses during pregnancy, or two doses prior to pregnancy plus a booster dose during pregnancy. COVID-19 vaccines were introduced in Türkiye in 2021; however, by 2022 they were no longer included in the routine vaccination schedule for pregnant women.

For analytical purposes, “vaccination during pregnancy” was defined as documentation of at least one vaccine dose administered during the index pregnancy, regardless of guideline-recommended timing. “Lifetime vaccination record” was defined as the presence of any documented vaccination entry prior to the index pregnancy in the national vaccination registry, without restriction to a specific age range or time period. In the absence of a documented vaccination record in the electronic systems, individuals were classified as not vaccinated for the corresponding vaccine. Individuals with no documented vaccination records at any time point were additionally classified as having no recorded lifetime vaccination history.

Vaccination records, including all doses administered both before and during pregnancy, were obtained from the family health centers’ electronic health record system and the National Vaccination Tracking System. In the analysis, vaccination rates were reported separately for doses received before pregnancy and those administered during pregnancy, while complete vaccination status was defined according to the doses required during the evaluated pregnancy.

The primary outcome of the study was vaccination during pregnancy, defined as having at least one documented vaccination record during the index pregnancy. This outcome was selected to reflect the clinical relevance of completion of recommended antenatal care visits in relation to maternal immunization in primary care. Secondary outcomes included the presence of a lifetime vaccination record and complete vaccination status for Td/Tdap, COVID-19 and complete immunization status for hepatitis B. Analyses of secondary outcomes were considered exploratory and were interpreted in relation to the primary outcome, with consideration given to the potential for multiple comparisons.

### Statistical Analysis

All statistical analyses were performed using IBM SPSS Statistics version 25.0 (IBM Corp., Armonk, NY, USA). Descriptive statistics were used to summarize the sociodemographic and clinical characteristics of the participants. Continuous variables were presented as mean ± standard deviation (SD), and categorical variables were expressed as frequencies and percentages. Although non-parametric tests were used for group comparisons due to non-normal distributions, continuous variables are presented as mean ± standard deviation for descriptive purposes only, to facilitate interpretability and comparability with previous studies in the maternal immunization literature.

The distribution of continuous variables was evaluated, and since data were not normally distributed, comparisons between two independent groups were conducted using the Mann–Whitney U test. Associations between categorical variables were analyzed using the Chi-square test. Comparisons of vaccination status before pregnancy and during pregnancy, representing paired binary data within the same individuals, were performed using McNemar’s test.

Multivariable binary logistic regression analyses were performed using separate models for each vaccination outcome. Vaccination during pregnancy (primary outcome), lifetime vaccination record, complete Td/Tdap vaccination, complete hepatitis B immunization status, and complete COVID-19 vaccination were modeled as dependent variables in individual analyses, with completion of recommended antenatal care visits included as the main explanatory variable. All models were constructed using an enter method, in which covariates were included simultaneously based on clinical relevance and prior evidence rather than univariable screening.

Prespecified covariates included maternal age, parity, presence of chronic disease, pregnancy-related medical conditions, and year of pregnancy outcome (2020–2022) to account for potential confounding, including pandemic-related temporal effects. Each outcome was analyzed in a separate adjusted model. The analytic sample size was 996 pregnancies for all outcomes, except for COVID-19 vaccination, which was restricted to pregnancies completed in 2021–2022 (n = 644).

Model diagnostics included assessment of multicollinearity among covariates and evaluation of overall model fit, and no major violations of model assumptions were identified. Results are reported as adjusted odds ratios (aORs) with 95% confidence intervals (CIs). A p-value of <0.05 was considered statistically significant.

Despite the retrospective design, the sample size was considered adequate for the planned multivariable logistic regression analyses. The number of outcome events was sufficient relative to the number of covariates included in each model, ensuring an acceptable events-per-parameter ratio and reducing the risk of model overfitting. Formal *post hoc* power calculations were not performed, as they are not recommended for observational studies; instead, the precision of the estimates is reflected in the reported confidence intervals.

### Ethical Considerations

Ethical approval for this study was obtained from the Ethics Committee Şişli Hamidiye Etfal Research and Training Hospital (Decision No: 4383, Date:30/04/2024) in accordance with the Declaration of Helsinki.

## Results

A total of 996 pregnant women were included in the study. The demographic and obstetric characteristics of the study population are summarized in Table 1.

**TABLE 1:** Demographic and obstetric characteristics of the study population (n = 996). Evaluation of Maternal Immunization Rates in Primary Care: A Retrospective Study from Selected Family Health Centers in Istanbul, Türkiye, 2020–2022.

Age-related variables	Mean ± SD (min, max)
Age	33.04 ± 5.48 (21–48)
Age at marriage	23.54 ± 4.78 (13–39)
Age at first pregnancy	24.94 ± 5.04 (15–41)
Obstetric history	
Gravidity	2.28 ± 1.34 (1–11)
Parity	1.83 ± 0.99 (0–7)
Abortions	0.33 ± 0.71 (0–5)
Living children	1.81 ± 0.97 (0–7)

SD, standart deviation.

A chronic disease was present in 138 participants (13.9%), most commonly hypothyroidism (n = 74, 54.0%), followed by asthma (n = 16, 11.7%). During pregnancy, 88 women (8.8%) were diagnosed with at least one pregnancy-related medical condition, again most frequently hypothyroidism (n = 54, 61.4%) followed by gestational diabetes mellitus (n = 17, 19.3%).

Before pregnancy, 524 participants (52.6%) had at least one vaccination record, most frequently Td/Tdap vaccines (n = 408, 41.0%) and hepatitis B vaccine (n = 37, 3.7%). COVID-19 vaccination was introduced in 2021; therefore, pre-pregnancy COVID-19 vaccination records were available only for pregnancies completed in 2021–2022 (n = 644), among whom 154 women (23.9%) had received at least one dose prior to conception. For all other vaccines, pre-pregnancy uptake was ≤0.3% (measles–mumps–rubella, PCV13, influenza, hepatitis A).

During pregnancy, 893 women (89.7%) had at least one vaccination record, most commonly Td/Tdap vaccines (n = 877, 98.2%) and hepatitis B vaccine (n = 40, 4.4%). COVID-19 vaccination was initiated in 2021; analyses restricted to pregnancies completed in 2021–2022 (n = 644) showed that 125 women (19.4%) received at least one dose of a COVID-19 vaccine during pregnancy.

Complete vaccination rates were 79.7% for Td/Tdap and 10.7% for COVID-19, while complete hepatitis B immunization status was observed in 9.8% of participants. The distribution of all vaccines and administered doses before and during pregnancy is presented in Table 2.

**TABLE 2:** Distribution of the number of vaccine doses before and during pregnancy among the same pregnancies (n = 996). Evaluation of Maternal Immunization Rates in Primary Care: A Retrospective Study from Selected Family Health Centers in Istanbul, Türkiye, 2020–2022^a^.

Vaccine	Dose category	Vaccination status before pregnancy (number of doses, n [%])	Vaccination status during pregnancy (number of doses, n [%])
Td	No documented record	582 (58.4%)	119 (11.9%)
	1 dose	194 (19.5%)	353 (35.5%)
	2 doses	146 (14.7%)	524 (52.6%)
	≥3 doses	72 (7.2%)	-
COVID-19^b^	No documented record	490 (76.1%)	519 (80.6%)
	1 dose	67 (10.4%)	63 (9.8%)
	2 doses	73 (11.3%)	62 (9.6%)
	≥3 doses	14 (2.2%)	-
Influenza	Yes	2 (0.2%)	5 (0.5%)
	No	994 (99.8%)	991 (99.5%)
Tdap	Yes	0 (0.0%)	2 (0.2%)
	No	996 (100%)	994 (99.8%)
Hepatitis B	No documented record	954 (95.2%)	956 (95.2%)
	1 dose	21 (2.1%)	20 (2.0%)
	2 doses	12 (1.2%)	28 (2.8%)
	3 doses	9 (0.9%)	-

Vaccination status before and during pregnancy refers to the same pregnancies assessed at two different time points.

^a^ Percentages are calculated using the total number of pregnancies in each column as the denominator (n/N, %).

^b^ COVID-19, vaccination data include pregnancies completed in 2021–2022 (n = 644), when the vaccine became available.

Td, tetanus–diphtheria; Tdap, tetanus–diphtheria–acellular pertussis.

McNemar’s test showed a statistically significant difference between vaccination status before pregnancy and during pregnancy (p < 0.001), with a higher proportion of women having at least one vaccination record during pregnancy. By pregnancy outcome year, 352 pregnancies (35.3%) ended in 2020, 309 (31.0%) in 2021, and 335 (33.7%) in 2022.

**FIGURE 2:**
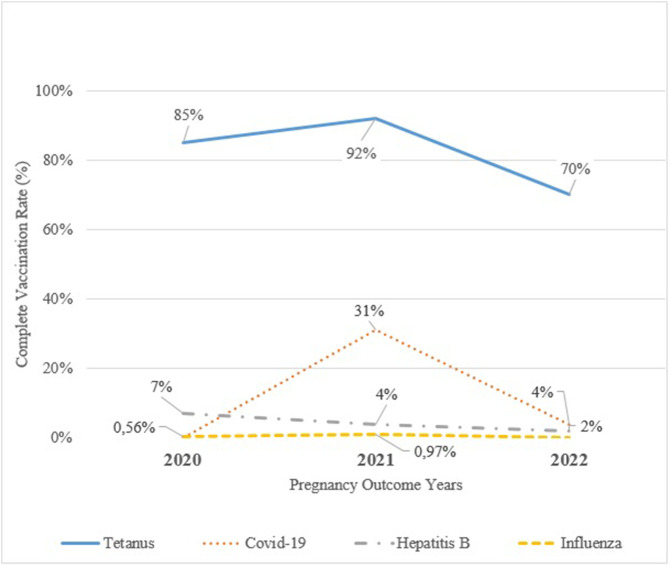
Trends in complete vaccination rates for tetanus, influenza, COVID-19 and complete immunization status for hepatitis B among pregnant women attending Family Health Centers (FHCs) in Istanbul, Türkiye, by pregnancy outcome year (2020–2022). COVID-19 vaccination data include only pregnancies completed in 2021–2022, when the vaccine was available. Evaluation of maternal immunization rates in primary care: a retrospective study from selected family health centers in Istanbul, Türkiye, 2020–2022.

The complete vaccination rates among pregnant women by year for Td/Tdap, COVID-19, and influenza, as well as complete hepatitis B immunization status, are presented in Figure 2. Td/Tdap vaccination consistently showed the highest coverage, rising from 85.8% in 2020 to 92.2% in 2021, followed by a decline to 70.4% in 2022. COVID-19 vaccination was highest in 2021 with 31.7% coverage but decreased to 4.5% in 2022. Complete Hepatitis B immunization status remained low throughout the study period, with rates of 7.4% in 2020, 4.2% in 2021, and 2.7% in 2022. Influenza vaccination was consistently below 1% and was not recorded at all in 2022.

When comparing Td/Tdap vaccination status with participants’ age, age at marriage, age at first pregnancy, gravidity, parity, number of abortions, and number of living children, a statistically significant association was found only between participants’ age and being fully vaccinated with Td (p = 0.016). Accordingly, the likelihood of being fully vaccinated with Td increased significantly with advancing maternal age.

Overall, 661 women (66.4%) completed the recommended antenatal care visits. However, women diagnosed with at least one medical condition during pregnancy were more frequently observed to have completed antenatal care visits (p = 0.003). No significant association was found between having a chronic disease before pregnancy and completion of recommended antenatal care visits (p = 0.783). Chronic disease status, whether before or during pregnancy, was not significantly associated with vaccination before or during pregnancy, nor with full vaccination against Td/Tdap, COVID-19 or complete immunization status for hepatitis B.

First-time pregnant women differed significantly from multiparous women in both pre-pregnancy and during-pregnancy vaccination records (p < 0.001 for both). Multiparous women were more likely to have pre-pregnancy vaccination records, while primigravidas were more likely to have received vaccinations during pregnancy.

The vaccination status during pregnancy, lifetime vaccination records, complete Td/Tdap vaccination, complete COVID-19 vaccination, and complete hepatitis B immunization status were compared between participants who completed all scheduled antenatal care visits and those who did not. The results are presented in Table 3.

**TABLE 3:** Comparison of vaccination status according to completion of antenatal care visits (n = 996). Evaluation of Maternal Immunization Rates in Primary Care: A Retrospective Study from Selected Family Health Centers in Istanbul, Türkiye, 2020–2022^a^.

Variables		Complete antenatal care n (%)	Incomplete antenatal care n (%)	p-value
Vaccination record during pregnancy	Yes	617 (93.3%)	276 (82.4%)	<0.001
	No	44 (6.7%)	59 (17.6%)	
Lifetime vaccination record	Yes	641 (97.0%)	304 (90.7%)	<0.001
	No	20 (3.0%)	31 (9.3%)	
Complete vaccination against tetanus	Yes	556 (84.1%)	238 (71.0%)	<0.001
	No	105 (15.9%)	97 (29.0%)	
Complete vaccination against COVID-19^b^	Yes	47 (10.9%)	22 (10.4%)	0.846
	No	385 (89.1%)	190 (89.6%)	
Immunization against Hepatitis B	Yes	75 (11.3%)	23 (6.9%)	0.025
	No	586 (88.7%)	312 (93.1%)	

^a^ Percentages are calculated using the total number of pregnancies in each column as the denominator (n/N, %).

^b^ COVID-19 vaccination data include pregnancies completed in 2021–2022 (n = 644), when the vaccine became available.

Table 4 shows that, in the adjusted binary logistic regression analyses, completion of recommended antenatal care visits was independently associated with higher odds of having a vaccination record during pregnancy (aOR 2.91, 95% CI 1.91–4.44, p < 0.001) and having a lifetime vaccination record (aOR 3.12, 95% CI 1.74–5.61, p < 0.001). Women with complete antenatal care visits were also twice as likely to be fully vaccinated against Td/Tdap (aOR 2.16, 95% CI 1.57–2.98, p < 0.001). Completion of recommended antenatal care visits showed a non-significant trend toward higher odds of complete hepatitis B immunization status (aOR 1.58, 95% CI 0.97–2.60, p = 0.068). No significant association was observed between antenatal care visit completion and complete COVID-19 vaccination during pregnancy in the 2021–2022 subcohort (aOR 0.99, 95% CI 0.57–1.71, p = 0.962).

**TABLE 4:** Adjusted associations between completion of antenatal care visits and maternal vaccination outcomes. Evaluation of maternal immunization rates in primary care: a retrospective study from selected family heath centers health centers in Istanbul, Türkiye, 2020–2022.

Variables^a^	aOR^c^	95% CI	p value
Vaccination record during pregnancy	2.91	1.91–4.44	<0.001
Lifetime vaccination record	3.12	1.74–5.61	<0.001
Complete vaccination against Tetanus	2.16	1.57–2.98	<0.001
Complete vaccination against COVID-19^b^	0.99	0.57–1.71	0.962
Complete Hepatitis B immunization status	1.58	0.97–2.60	0.068

Multivariable binary logistic regression analysis.

All analyses were based on n = 996 pregnancies unless otherwise specified.

^a^ Each outcome represents a separate adjusted binary logistic regression model with completion of antenatal care visit as the main explanatory variable.

^b^ COVID-19 vaccination models were restricted to pregnancies completed in 2021–2022 (n = 644).

^c^ Reference categories were: incomplete antenatal care visits (for the main explanatory variable), no vaccination (for all vaccination outcomes), and absence of chronic disease or pregnancy-related medical conditions, as applicable.

All models were adjusted for maternal age, parity, chronic disease, pregnancy-related medical conditions, and year of pregnancy outcome (2020–2022).

aOR, adjusted odds ratio; CI, confidence interval.

## Discussion

This retrospective study evaluated maternal vaccination coverage across eight family medicine centers in Istanbul, Türkiye. Overall, while 89.7% of pregnant women received at least one vaccine, complete vaccination rates varied starkly: 79.7% for Td/Tdap, 10.7% for COVID-19, 9.8% for Hepatitis B, and less than 1% for influenza. In adjusted analyses, completion of recommended antenatal care visits was independently associated with significantly higher odds of having any vaccination record (aOR 2.91, 95% CI 1.91–4.44) and complete Td/Tdap vaccination (aOR 2.16, 95% CI 1.57–2.98) whereas no such significant association was observed for COVID-19 or Hepatitis B.

Completion of recommended antenatal care visits, considered a marker of continuity of care and health-seeking behavior in this study, was associated with higher odds of maternal vaccination uptake during pregnancy, suggesting a potential link between structured primary care follow-up and maternal immunization. In Türkiye, Family Health Centers constitute the primary setting where maternal vaccination counseling and access to vaccines are routinely provided. Accordingly, women who maintain regular contact with primary care services may be more likely to encounter opportunities for vaccination recommendation and delivery within routine antenatal care. From this perspective, the observed association likely reflects the role of continuity of primary care in maternal immunization.

While coverage of tetanus toxoid–containing vaccines, predominantly Td in this cohort, was relatively high, the near-absence of influenza vaccination and low uptake of Hepatitis B and COVID-19 vaccines highlight substantial missed opportunities. In Türkiye, reported coverage rates for tetanus toxoid–containing vaccines among pregnant women have ranged between 57% and 68% over the past 5 years [20]. Although direct comparisons are limited by differences in definitions and data sources, these findings collectively suggest that maternal immunization efforts in Türkiye have primarily focused on tetanus-containing vaccines, with substantial missed opportunities for improving coverage of other recommended maternal vaccines.

Among the participants, 66.4% completed recommended antenatal care visits, and this group had higher Td/Tdap vaccination coverage compared to those with incomplete antenatal care visit. These findings highlight the pivotal role of continuity in antenatal care as a marker of health-seeking behavior associated with maternal immunization. Our logistic regression analysis showed that women who completed the recommended antenatal care had higher odds of being fully vaccinated against Td/Tdap and of having at least one documented vaccination record during pregnancy. This is consistent with evidence from both Türkiye and other settings, where structured, repeated contact with healthcare providers has been associated with higher vaccine acceptance, potentially reflecting increased opportunities for counseling, recommendation, and administration [15–18].

The uptake of tetanus toxoid–containing vaccines—predominantly Td in this cohort- (79.7% fully vaccinated in our study) appears relatively high; however, this figure should be interpreted in context. According to the World Health Organization (WHO), at least 80% of pregnant women in every district must be fully vaccinated during pregnancy to achieve and sustain maternal and neonatal tetanus elimination (MNTE) [21]. In Türkiye, the Ministry of Health launched a national MNTE program in 1994, and tetanus toxoid-containing vaccines have since been systematically integrated into antenatal care and delivered free of charge through FHCs [13, 14, 22, 23]. Despite this infrastructure, coverage in our sample only narrowly approached the WHO threshold, and national reporting shows even lower rates-68% in 2022-placing Türkiye behind several other low- and middle-income countries [24]. These findings suggest that, although tetanus-focused maternal immunization has been relatively successful, gaps in consistent antenatal care delivery and implementation may still limit full coverage at the population level.

In contrast, the extremely low uptake of Tdap vaccine (0.2%) is likely related to its exclusion from the national immunization schedule and lack of reimbursement during the study period. Although international guidelines recommend Tdap in every pregnancy, Türkiye incorporated routine Tdap vaccination into the national program only after 2025 [12].

In the 2021–2022 subcohort, COVID-19 vaccination uptake was 19.4%. Notably, the highest uptake occurred in 2021—the first year COVID-19 vaccines became available for pregnant women—which may reflect the immediacy of the pandemic, heightened risk perception, along with evolving national recommendations and changes in vaccine access and delivery and intensive public health messaging may have created a temporary window of higher acceptance [25, 26]. However, the observed fluctuations in vaccination uptake are likely to reflect a combination of heightened risk perception during the pandemic and concurrent structural changes, such as evolving national recommendations and variations in vaccine availability for pregnant women.

Similarly, the peaks observed in tetanus toxoid–containing vaccination (Td/Tdap) and hepatitis B immunization rates in 2020, followed by a subsequent decline, may be related to combined influence of pandemic-related risk perception and concurrent changes in health system priorities and vaccination policies. Enhanced public awareness activities and heightened health concerns during the acute phase of the pandemic may have contributed to these temporary increases.

In contrast, influenza vaccination did not follow the same pattern. One possible explanation is that during the pandemic, the overwhelming focus on COVID-19, coupled with the reduced circulation of seasonal influenza viruses due to widespread preventive measures, may have been associated with an even lower demand for influenza vaccination among pregnant women. This reflects the already marginal uptake of maternal influenza vaccination in Türkiye, which remained unaffected or further diminished during the pandemic.

When considering the broader impact of the pandemic on immunization, our findings are consistent with the patterns reported globally: during the acute phase of the pandemic, vaccine uptake was observed to increase due to heightened risk perception, but this effect was not sustained once the immediate threat subsided. A similar “pandemic effect”, likely reflecting a combination of heightened risk perception, policy changes, and vaccine availability, was documented during the 2009–2010 H1N1 influenza pandemic, when maternal influenza vaccination rates in Türkiye reached their historical peak, with two separate studies reporting rates of 9.1% and 8.9%, respectively [27, 28]. Although these figures were still considerably lower than those in high-income countries such as the United States (45.7%) during the same period [29], they were markedly higher than non-pandemic baseline levels in Türkiye, which often fall below 1% [30].

This phenomenon was not limited to maternal immunization. During the COVID-19 pandemic, adult immunization coverage for other vaccines such as the pneumococcal conjugate vaccine (PCV13) was observed to increase, coincide with heightened risk perception and intensified public health messaging. For instance, in Hong Kong, coverage among older adults rose from 17.3% pre-pandemic to 28.3% during the pandemic and 35.5% post-pandemic [31]. Similarly, in Türkiye, the number of adult vaccine doses administered in primary care centers more than doubled during the pandemic period compared with the previous year [32]. However, sustaining these gains in routine, non-pandemic settings appears to remain a major challenge.

Evidence from multiple settings indicates that the most consistent factor associated with maternal vaccine acceptance-both during and outside of pandemic periods-is a strong, clear, and timely recommendation from a trusted healthcare provider [15–18]. However, provider recommendation alone may not be sufficient if not supported by structural enablers, such as the systematic integration of all recommended maternal vaccines into antenatal care protocols, standing orders for vaccination, and vaccine availability at the point of care [25, 26]. In the Turkish context, while tetanus toxoid–containing (Td/Tdap) vaccination benefits from these integrated mechanisms, hepatitis B, influenza and COVID-19, have not been equally embedded in routine antenatal care visits, which may contribute to missed opportunities. Addressing these systemic gaps appears important for translating temporary gains achieved during pandemics into sustained improvements in maternal immunization coverage.

International evidence supports this view, highlighting several effective strategies that have been associated with improved maternal immunization uptake. These include patient education and tailored communication—especially addressing safety concerns and perceived benefits [33]; provider education and reminder systems [34]; digital tools such as SMS alerts and web-based platforms [34, 35]; and policy-level enablers such as mandatory documentation in antenatal records, standing orders, and provider incentives [36]. Two of the most consistently reported predictors of maternal vaccination are trusted healthcare provider advice and the integration of vaccines into routine antenatal care visits [17, 18].

### Limitations

This study has several limitations. Its retrospective design relies on existing health records, which may be subject to incomplete or inaccurate data entry, although vaccination information was verified through the National Vaccination Tracking System to minimize reporting bias. Because of the retrospective observational design, the associations observed between completion of antenatal care visits and maternal vaccination outcomes cannot be interpreted as causal relationships. Completion of antenatal care visits was used as a proxy indicator of continuity of care and health-seeking behavior rather than a direct exposure. In addition, vaccinations documented only on personal vaccination cards and not entered into electronic systems may not have been captured, potentially resulting in non-differential misclassification and underestimation of true vaccination coverage.

Second, the analysis was limited to Family Health Centers within a single metropolitan region, which may restrict generalizability to rural or underserved settings with different healthcare access patterns. Moreover, variability across Family Health Centers in catchment population size, socioeconomic characteristics, migrant density, and service capacity may have influenced both antenatal care utilization and vaccination recording practices.

Because of the low uptake of non-tetanus vaccines, some subgroup analyses could not be performed. Also exclusion of pregnancies ending in stillbirth or abortion, as well as those with incomplete records, may introduce selection bias, particularly if these groups differ in healthcare utilization or vaccination behavior. However, this exclusion was methodologically necessary, as pregnancies that ended prematurely may not have had sufficient opportunity to complete vaccines recommended during pregnancy, which could otherwise have led to misclassification of vaccination status. However, these exclusions may have introduced selection bias if excluded pregnancies differed systematically from included pregnancies in terms of healthcare utilization patterns or vaccination behavior. Nevertheless, this consideration should be taken into account when interpreting the findings.

Hepatitis B status was defined based on documented vaccine doses and/or anti-HBs seropositivity, reflecting immune status rather than confirmed vaccination history alone. Anti-HBs positivity does not allow differentiation between vaccine-induced and naturally acquired immunity, nor precise determination of the timing of immunity acquisition relative to pregnancy. In addition, interpreting the absence of a documented record as non-vaccination may have resulted in misclassification, particularly among women vaccinated historically before full integration of electronic registries. Such potential non-differential misclassification would be expected to bias association estimates toward the null and may partly explain the non-significant findings observed for hepatitis B.

Because uptake of several non-tetanus vaccines was low, some subgroup analyses could not be performed, limiting the ability to explore effect modification across specific vaccine types. Multiple vaccination outcomes were evaluated in this study. Although these outcomes were pre-specified and clinically relevant, the possibility of inflated type I error due to multiple comparisons should be considered, particularly for secondary outcomes.

Finally, residual confounding cannot be excluded. Although regression models were adjusted for key maternal and pregnancy-related characteristics, unmeasured factors such as socioeconomic status, education level, health literacy, and provider-level practices may have influenced both antenatal care attendance and vaccination outcomes. In addition, the study did not assess reasons for vaccine acceptance or refusal, which would require qualitative or prospective designs.

### Conclusion

Maternal vaccination coverage in Türkiye remains below target levels, except for tetanus toxoid–containing (Td/Tdap). Greater integration of all recommended maternal vaccines—particularly influenza and hepatitis B—into national antenatal care visits may be associated with improved coverage. Ensuring that every antenatal care visit includes an opportunity for vaccine counseling and administration, combined Digital reminder systems, audit-feedback mechanisms, and pregnancy education programs could further support sustained improvements. Furthermore, establishing nationwide monitoring of maternal vaccination indicators may enhance data quality and help inform policy updates. Future research should focus on identifying barriers to non-tetanus vaccine acceptance and on evaluating the effectiveness of targeted interventions—such as provider prompts, electronic tracking, and patient education—in relation to optimizing maternal immunization in Türkiye.
